# Profiling cytotoxic microRNAs in pediatric and adult glioblastoma cells by high-content screening, identification, and validation of miR-1300

**DOI:** 10.1038/s41388-020-1360-y

**Published:** 2020-06-17

**Authors:** M. Boissinot, H. King, M. Adams, J. Higgins, G. Shaw, T. A. Ward, L. P. Steele, D. Tams, R. Morton, E. Polson, B. da Silva, A. Droop, J. L. Hayes, H. Martin, P. Laslo, E. Morrison, D. C. Tomlinson, H. Wurdak, J. Bond, S. E. Lawler, S. C. Short

**Affiliations:** 1 Radiation Biology and Therapy Group, Leeds Institute of Medical Research, University of Leeds, St James’s Hospital, Leeds, LS9 7TF UK; 2 BioScreening Technology Group, University of Leeds, St James’s Hospital, Leeds, LS9 7TF UK; 3 Roslin Cell Sciences, Babraham, Cambridge, CB22 3AT UK; 4 Stem Cells and Brain Tumour Research Group, Leeds Institute of Medical Research, University of Leeds, St James’s Hospital, Leeds, LS9 7TF UK; 5 MRC Medical Bioinformatics Centre, University of Leeds, Clarendon Way, Leeds, LS2 9NL UK; 6 School of Public Health, University of California Berkeley, Berkeley, CA 94720 USA; 7 School of Molecular and Cellular Biology, Faculty of Biological Sciences, University of Leeds, Leeds, LS2 9JT UK; 8 Myeloid Differentiation Group, Leeds Institute of Medical Research, University of Leeds, St James’s Hospital, Leeds, LS9 7TF UK; 9 Cell Biology Research Group, Leeds Institute of Medical Research, University of Leeds, St James’s Hospital, Leeds, LS9 7TF UK; 10 Microcephaly and Neurogenesis Research Group, Leeds Institute of Medical Research, University of Leeds, St James’s Hospital, Leeds, LS9 7TF UK; 11 Harvey Cushing Neurooncology Laboratories, Department of Neurosurgery, Brigham and Women’s Hospital, Harvard Medical School, Boston, MA 02115 USA; 12 St James’s Institute of Oncology and Leeds Institute of Medical Research, University of Leeds, St James’s Hospital, Leeds, LS9 7TF UK

**Keywords:** Oncogenes, Mitosis, Non-coding RNAs

## Abstract

MicroRNAs play an important role in the regulation of mRNA translation and have therapeutic potential in cancer and other diseases. To profile the landscape of microRNAs with significant cytotoxicity in the context of glioblastoma (GBM), we performed a high-throughput screen in adult and pediatric GBM cells using a synthetic oligonucleotide library representing all known human microRNAs. Bioinformatics analysis was used to refine this list and the top seven microRNAs were validated in a larger panel of GBM cells using state-of-the-art in vitro assays. The cytotoxic effect of our most relevant candidate was assessed in a preclinical model. Our screen identified ~100 significantly cytotoxic microRNAs with 70% concordance between cell lines. MicroRNA-1300 (miR-1300) was the most potent and robust candidate. We observed a striking binucleated phenotype in miR-1300 transfected cells due to cytokinesis failure followed by apoptosis. This was also observed in two stem-like patient-derived cultures. We identified the physiological role of miR-1300 as a regulator of endomitosis in megakaryocyte differentiation where blockade of cytokinesis is an essential step. In GBM cells, where miR-1300 is normally not expressed, the oncogene Epithelial Cell Transforming 2 (ECT2) was validated as a direct key target. ECT2 siRNA phenocopied the effects of miR-1300, and ECT2 overexpression led to rescue of miR-1300 induced binucleation. We showed that ectopic expression of miR-1300 led to decreased tumor growth in an orthotopic GBM model. Our screen provides a resource for the neuro-oncology community and identified miR-1300 as a novel regulator of endomitosis with translatable potential for therapeutic application.

## Introduction

MicroRNAs are small 22–24 nt single-stranded noncoding RNAs that function by reducing the translation of target mRNAs. In glioblastoma (GBM), they have been shown to play roles in proliferation, invasion, and stemness, suggesting that microRNAs and their downstream pathways may represent potent therapeutic targets [1–6]. There are an increasing number of microRNA mimics and inhibitors in preclinical and early clinical development as cancer therapies, including one for patients with solid tumors using an oligonucleotide mimic of microRNA-34 (MRX34, NCT01829971) [7–10]. Recent preclinical studies showed efficacy of a microRNA expressing therapeutic vector in GBM [11]. MicroRNA-10b expression has been measured in a clinical trial (NCT01849952) to assess its use as a prognostic and diagnostic biomarker. An inhibitor of miR-10b is also currently at the preclinical development stage (Regulus Therapeutics Inc. and [12]).

Current approaches for microRNA studies in GBM mainly involve endogenous microRNA expression profiles coupled with bioinformatic analysis and target identification to link the landscape of microRNA expression to GBM biology and disease outcome [13–15]. Other functional studies have focused on small numbers of microRNAs and very few large-scale functional studies have been performed in GBM [16].

To assess the landscape of potential cytotoxic microRNAs in GBM we decided on a global approach by performing a large-scale functional screen. We used a microRNA mimic oligonucleotide library combined with a high-throughput imaging platform to identify microRNAs that significantly impaired proliferation and/or survival of GBM cells. This approach highlighted microRNA-1300 as a candidate for more detailed characterization. We found that miR-1300 is not typically expressed in GBM, and that ectopic expression of its mature form following transfection consistently caused a G2/M cell cycle arrest followed by apoptosis. Further validation showed that forced expression of miR-1300 in GBM cells caused cytokinesis failure and that the oncogene Epithelial Cell Transforming 2 (ECT2) is one of the direct targets of miR-1300 involved in this phenotype. This, in turn, led us to identify the key physiological need for the finely tuned, cell, and stage-specific, expression of miR-1300 during endomitosis in platelet formation from megakaryocytes [17–19]. Finally, we confirmed the cytotoxicity of ectopic expression of miR-1300 in a preclinical orthotopic model of GBM. Taken together, our study not only provides an encompassing profile of cytotoxic microRNAs toward adult and pediatric GBM cells but also identifies miR-1300 as a uniquely specific tool with a potential therapeutic window for combination with current standard therapy in GBM. Our dataset will provide a useful resource for researchers in the field with an interest in the therapeutic application of microRNAs.

## Results

### A high-throughput screen identifies microRNAs with cytotoxic activity in GBM

We profiled cytotoxic microRNAs in adult and pediatric GBM cells using a high-throughput high-content screen based on a library that encompassed mimics of the mature form of all annotated microRNAs based on miRBase v16.0 at the time. The screen was performed in two established GBM cell lines: U251 (adult GBM) and KNS42 (pediatric GBM). A schematic of the screen is shown in Supplementary Fig. 1. The chosen primary end-point read out was a decrease in cell number assessed by automated nuclei counting at 72 h post transfection.

The candidate hit list for KNS42 and U251 cells contained 83 and 304 hits, respectively (see Supplementary Tables 1 and 2 for the complete screen datasets). The U251 list was re-analyzed using a < −3 standard deviation cutoff (see “Methods” for details), which gave 111 hits to consider for further analysis. Based on the *Z*-score analysis, we initially observed a 70% overlap between the candidate lists for each cell line (Supplementary Table 3) and focused further investigations on those microRNAs. We utilized the online microRNA databases miRBase [20–24] and Targetscan [25–31], as well as PubMed (National Center for Biotechnology Information, National Library of Medicine, Bethesda, MD, USA) to gather available information for each of the “hit” microRNAs: chromosomal location, main validated and predicted target genes, associated functions and role in disease. This approach shortlisted 18 candidate microRNAs. The second level of analysis took into consideration the strength of the *Z*-score, together with information focused on seed sequence family and the association of the target genes with cell proliferation and/or cell death and led to the selection of seven microRNAs for validation in cell-based assays. The endogenous levels of expression of those seven microRNAs were verified in our established cell line models and found mostly not expressed in our cells (Supplementary Fig. 2). We applied the conditions of the primary screen to four established GBM cell lines (U251, KNS42, LN229, and U373), confirming statistically significant cytotoxicity following transfection of all seven mimic-microRNAs (Supplementary Fig. 3a).

In order to investigate whether cytotoxicity was due to programmed cell death, we measured the number of cleaved caspase-3 foci by immunofluorescence. Interestingly, only transfection with the mimic for miR-1300 led to the cleavage of caspase-3 and apoptotic cell death (Supplementary Fig. 3b, c).

We then focused further on characterizing the role and mechanism of action of miR-1300; based on its high *Z*-scores (*Z*-score = −2.98 in KNS42 and *Z*-score = −5.14 in U251), and its ability to induce apoptosis. MiR-1300 mature and precursor sequences as well as alignment to the human genome are described in Supplementary Table 4.

### MiR-1300 induces cytokinesis failure, cell cycle arrest and apoptosis in GBM cell lines and patient-derived GBM

**Fig. 1:**
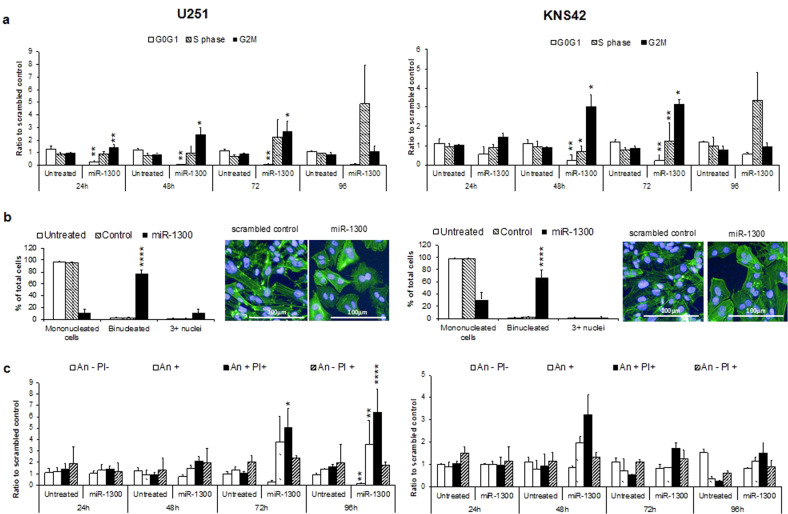
Effect of miR-1300 expression on proliferation and cell death in U251 and KNS42 cell lines. **a** Cell cycle time course analyzed by flow cytometry using propidium iodide (PI) loading as a measure of DNA content. **b** Binucleation phenotype scoring following following 72 h of treatment and staining with DAPI and Phalloidin Alexafluor488 (Actin) and the corresponding representative image. All experiments were performed in triplicate. The results are normalized to a scrambled-mimic control. **c** Cell death measured by flow cytometry where Annexin negative (An−)/PI negative (PI−) = live cells, An+ = early-mid apoptotic cells, An+ PI+ = mid-late apoptotic cells and An− PI + = necrotic cells. Statistical significance is expressed as follows: *p < 0.05; **p < 0.01; ***p < 0.001; ****p < 0.0001.

Flow cytometry-based assays were used to measure the effect of ectopic expression of miR-1300 in its mature form (MIMIC) on cell cycle and cell death over time. Expression of miR-1300 induced a cell cycle significant block in G2/M at 24 h in U251 and 48 h in KNS42 cells (Fig. 1a) with a multinucleation phenotype (Fig. 1b), rapidly followed by the onset of apoptosis (Fig. 1c). In miR-1300 treated U251 cells there was a > 60% reduction in cells in G0/G1 with a 1.5-fold increase in G2/M cells at 24 h compared with the scrambled control (*p* = 0.0079**, Fig. 1a). This G2/M arrest became more pronounced at 48 h (~2.5-fold change, *p* < 0.0335*) and 72 h (threefold change, *p* < 0.0485*) but was no longer apparent at 96 h following high levels of apoptosis (Fig. 1c) with >20% reduction in the number of live cells and approximately three- and sixfold increase of cells in early-mid and mid-late apoptosis (*p* = 0.0012** and *p* < 0.0001****, respectively, Fig. 1b). Similar changes in the cell cycle profile were observed in KNS42 cells with an approximately four- and approximately threefold increase in cells in G2/M at 48 and 72 h (*p* = 0.017* and *p* = 0.009**), respectively. Analysis of immunofluorescently stained cells from the screen showed that cell cycle arrest observed by flow cytometry occurred during mitosis, where 72 h post transfection, ectopic expression of miR-1300 caused ~80% and >60% increase in binucleated cells in U251 and KNS42, respectively (*p* < 0.0001****; Fig. 1c). The same phenotype was observed in LN229 and U373 GBM cell lines (Supplementary Fig. 4). The binucleation we observed (Fig. 1c) suggested that cell cycle arrest took place after telophase and was representative of cytokinesis failure. Live cell imaging confirmed that both KNS42 (Supplementary Movie 1A) and U251 cells (Supplementary Movie 1B) transfected with miR-1300 initiated mitosis normally but failed to complete the final stages of cytokinesis, resulting in the formation of multinucleated cells.

**Fig. 2:**
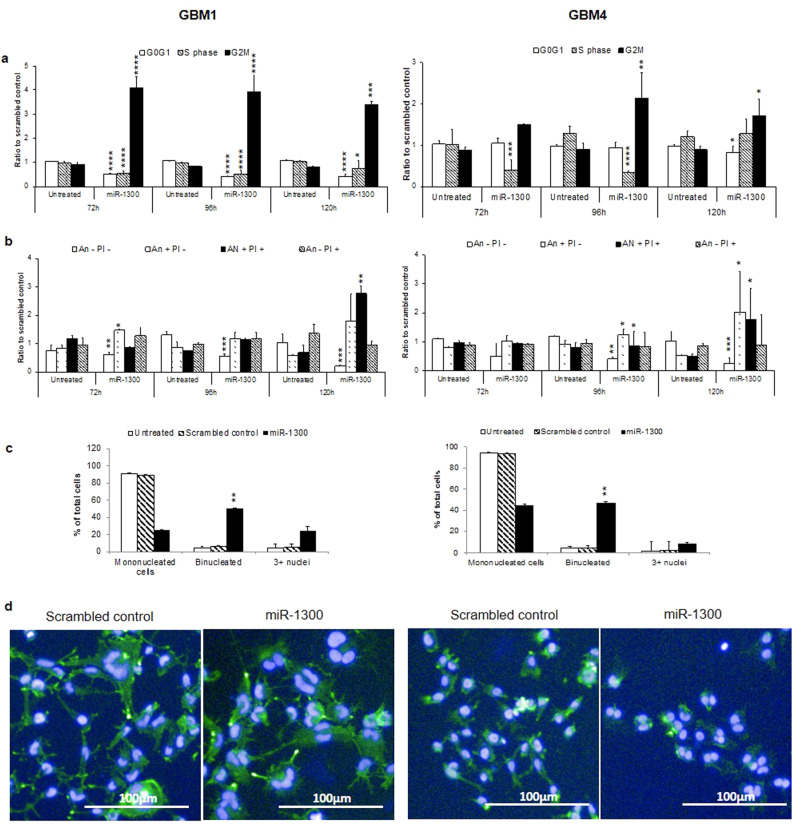
Effect of miR-1300 expression on proliferation and cell death in GSC cultures GBM1 and GBM4. **a** Cell cycle time course analyzed by flow cytometry using propidium iodide (PI) loading as a measure of DNA content. **b** Cell death measured by flow cytometry where Annexin negative (An−), PI negative (PI−) = live cells, An+ PI− = early-mid apoptotic cells, An+ PI+ = mid-late apoptotic cells and An− PI+ = necrotic cells. **c**, **d** Binucleation phenotype scoring following staining with DAPI and Phalloidin Alexafluor488 (Actin) (3–6 images per condition, representing at least 100 cells) was performed on images taken on the Operetta imaging platform (×10 objective). All experiments were performed in triplicate. The results are normalized to a scrambled-mimic control. Statistical significance is expressed as follows: *p < 0.05; **p < 0.01; ***p < 0.001; ****p < 0.0001.

Next, we sought to confirm that this phenotype was also observed in patient-derived glioma stem-like cells (GSCs) as a more representative in vitro model. In two previously characterized GSCs, GBM1 and GBM4 [32, 33] new time courses for the flow cytometry assays were performed at 72, 96, and 120 h to reflect the slower division rate of the patient-derived cells. Similarly, imaging experiments were replicated with binucleation scoring at 96 h. Transfection of GBM1 with mimic-miR-1300 caused a profound G2/M phase block with a fourfold increase in the proportion of cells in G2/M phase at 72, 96, and 120 h compared with cells transfected with the scrambled-mimic control (Fig. 2a). Transfection with mimic-miR-1300 also caused a twofold reduction in the number of cells in G0/G1 and S-phase (Fig. 2a). This G2/M arrest led to an overall reduction in live cells of ~40%, 45%, and 80% at 72, 96, and 120 h and a significant increase (*p* < 0.01 and *p* < 0.05 in GBM1 and GBM4, respectively) in apoptotic cells at 120 h (Fig. 2b). Similar results were observed in GBM4 GSCs with miR-1300 causing a 65% reduction in cells in S-phase at 72 h, which was maintained at 96 h. At 96 h there was an increase in cells on G2/M phase by fourfold for GBM1 and by twofold for GBM4 (Fig. 2a). At 96 h, miR-1300 transfection resulted in a ~40% reduction in live cells and an increase of around 20% in early-mid apoptotic GBM1 cells. By 120 h there was a ~75% decrease in live cells and a 1.5-fold increase in early-mid apoptotic cells for both GBM1 and GBM4, and 2.5 and 1.5-fold increase in mid-late apoptotic cells for GBM1 and GBM4, respectively (Fig. 2b). Figure 2c, d shows the effect of miR-1300 on the number of bi- and multinucleated cells. As with the established cell lines, miR-1300 caused a decrease in mono-nucleated cells of ~90% in both GBM1 and GBM4 and a significant increase (~50%) in binucleated cells for both GSCs (Fig. 2c, d), (*p* = 0.0055** and *p* = 0.0081**, respectively).

Taken together, this initial characterization showed that ectopic expression of miR-1300 consistently led to cytokinesis failure, measured by cell cycle arrest in G2/M phase and manifested by a striking binucleated phenotype followed by the onset of apoptosis as documented by caspase-3 cleavage (Supplementary Fig. 3b, c) and expression of Annexin V/PI (Figs. 1b and 2b). This was confirmed both in established GBM cells and in patient-derived GSCs.

### Identification of miR-1300 target genes

**Fig. 3:**
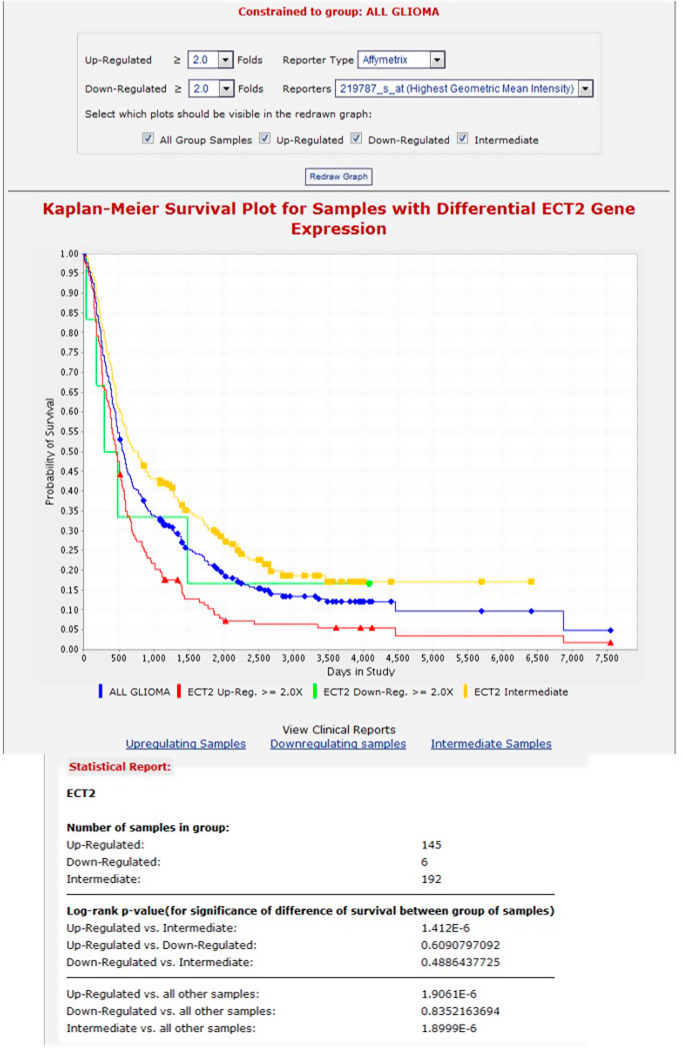
The Rembrandt database was used to search for a correlation between the levels of ECT2 mRNA and survival in glioma patients. We found a significant difference in survival between glioma patients with higher levels of ECT2 mRNA and patients with intermediate ECT2 mRNA levels. This was also true when compared to all glioma samples in the database.

Since there are no validated target genes for miR-1300, we used the list of predicted targets extracted from the online database TargetScan v5.2 [29]. This contains 3327 target genes predicted to be targeted by miR-1300 irrespective of the presence of conserved sites (the seed sequence for miR-1300 is poorly conserved across species). This list was analyzed using Metacore gene software (Thomson–Reuters) and cross-referenced using AmiGO [34] for “cytokinesis” as a Gene Ontology term. This identified which of the miR-1300 target genes had the highest potential involvement in the observed cytokinesis failure, resulting in a list of 21 potential target genes (Supplementary Table 5). Based on the characteristic binucleation seen in our phenotype, the Targetscan prediction score, and the literature, we chose to focus on the guanine nucleotide exchange factor (GEF) *ECT2* gene as our target of interest for initial validation. Interestingly, *ECT2* has been previously described as an oncogene and has been shown to contribute to the invasive behavior of GBM cells [35–39]. Analysis of survival data in gliomas in the Rembrandt (REpository for Molecular BRAin Neoplasia DaTa) dataset showed that patients with increased levels of ECT2 mRNA have a lower survival than those with intermediate levels (Fig. 3). *ECT2* plays a crucial role in cytokinesis through activation of the small GTPase RhoA, a key protein in the formation of the mitotic cleavage furrow during cytokinesis [17, 18]. Also, treatment of cells with RhoA inhibitors caused a binucleated phenotype similar to that observed in the GBM cell lines following transfection with miR-1300 [40]. Further, ECT2 depletion has been shown to lead to cytokinesis failure by impairment of cleavage furrow formation [41].

Using Real-Time qPCR in a panel of five patient-derived GSCs, an inverse relation between *ECT2* mRNA and miR-1300 expression was observed (Supplementary Fig. 5a). This is consistent with the frequency of multinuclear cells observed during cell culture (Supplementary Fig. 5b). Together, these data implicate ECT2 as a miR-1300 target that may play a role in mediating its effects on GBM cells.

### ECT2 is a direct target of miR-1300

**Fig. 4:**
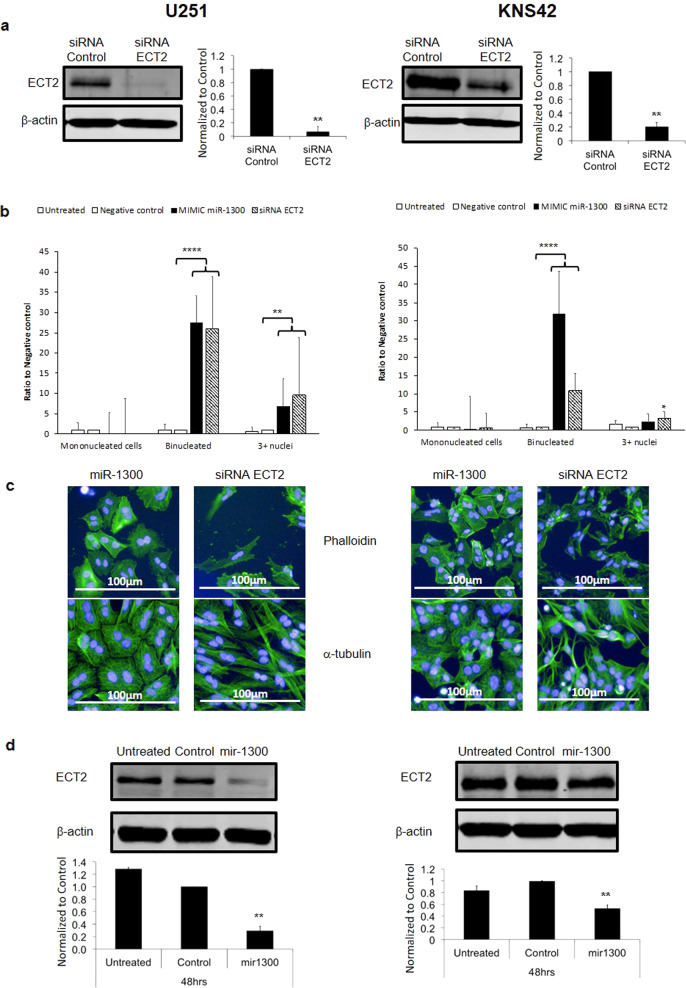
Reduced ECT2 levels in response to miR-1300 expression is associated with cytokinesis failure in U251 and KNS42 cell lines. **a** Confirmation of ECT2 knockdown at the protein level (by WB) following transfection with ECT2 siRNA smartpool. All experiments were performed in triplicate. The results were normalized to a scrambled-mimic control. **b** Transfection with ECT2 siRNA leads to an increase in binucleated cells. Binucleation phenotype scoring (3–6 fields of view (FOV) per condition, representing at least 100 cells) was performed on images taken on the Operetta imaging platform (×10 objective). **c** Immunofluorescence images comparing the actin and alpha-tubulin staining in U251 and KNS42 cells 72 h following transfection with 100 nM of either miR-1300 or ECT2 siRNA showing both the common effect on binucleation (DAPI, blue) and the comparative differences in actin (Phalloidin) and a-tubulin structures (Alexafluor488, green). Scale bar = 100 mm. **d** Ectopic expression of miR-1300 leads to decreased expression of ECT2 at the protein level. Statistical significance is expressed as follows: **p < 0.01; ****p < 0.0001. NB: Blot images were taken at 48 h time point since at 72 h, cells in control conditions have reached confluence, and were no longer expressing ECT2 as they are not dividing.

To validate *ECT2* as a direct target of miR-1300, U251 and KNS42 cells were transfected with siRNAs directed against *ECT2* (~95% reduction in U251 and ~80% reduction in KNS42 (Fig. 4a and Supplementary Fig. 6b)). *ECT2* siRNA-mediated knockdown replicated the binucleated phenotype induced by miR-1300 observed for mimic-miR-1300 (Fig. 4b, c, left hand side). Transfection of U251 cells with miR-1300 caused ~30% and 7% increase in bi- and multinuclear cells, respectively. Consistent with identification of ECT2 as a miR-1300 target, transfection with miR-1300 induced a decrease in the expression of ECT2 of ~70% and 50% in U251 and KNS42 cells, respectively (Fig. 4d and Supplementary Fig. 6a); siRNA-mediated knockdown of *ECT2* caused an increase in bi- and multinucleated cells of ~40% and 15%, respectively. A similar trend was observed in KNS42 cells (Fig. 4b, c, right hand side). Western blotting experiments confirmed that transfection with miR-1300 induces a decrease in the expression of ECT2 of ~70% and 50% in U251 and KNS42 cells, respectively (Fig. 4d and Supplementary Fig. 6a). Experiments in the patient-derived GSCs also showed that individual *ECT2* siRNAs led to a significant increase in binucleated cells (Supplementary Fig. 7). Structural differences in actin and tubulin between miR-1300 expressing and *ECT2* knockdown cells were observed by immunofluorescence (Fig. 4b). This suggests that other miR-1300 target genes, likely from the list of 21 targets we previously identified (Supplementary Table 5), affect its downstream phenotype.

**Fig. 5:**
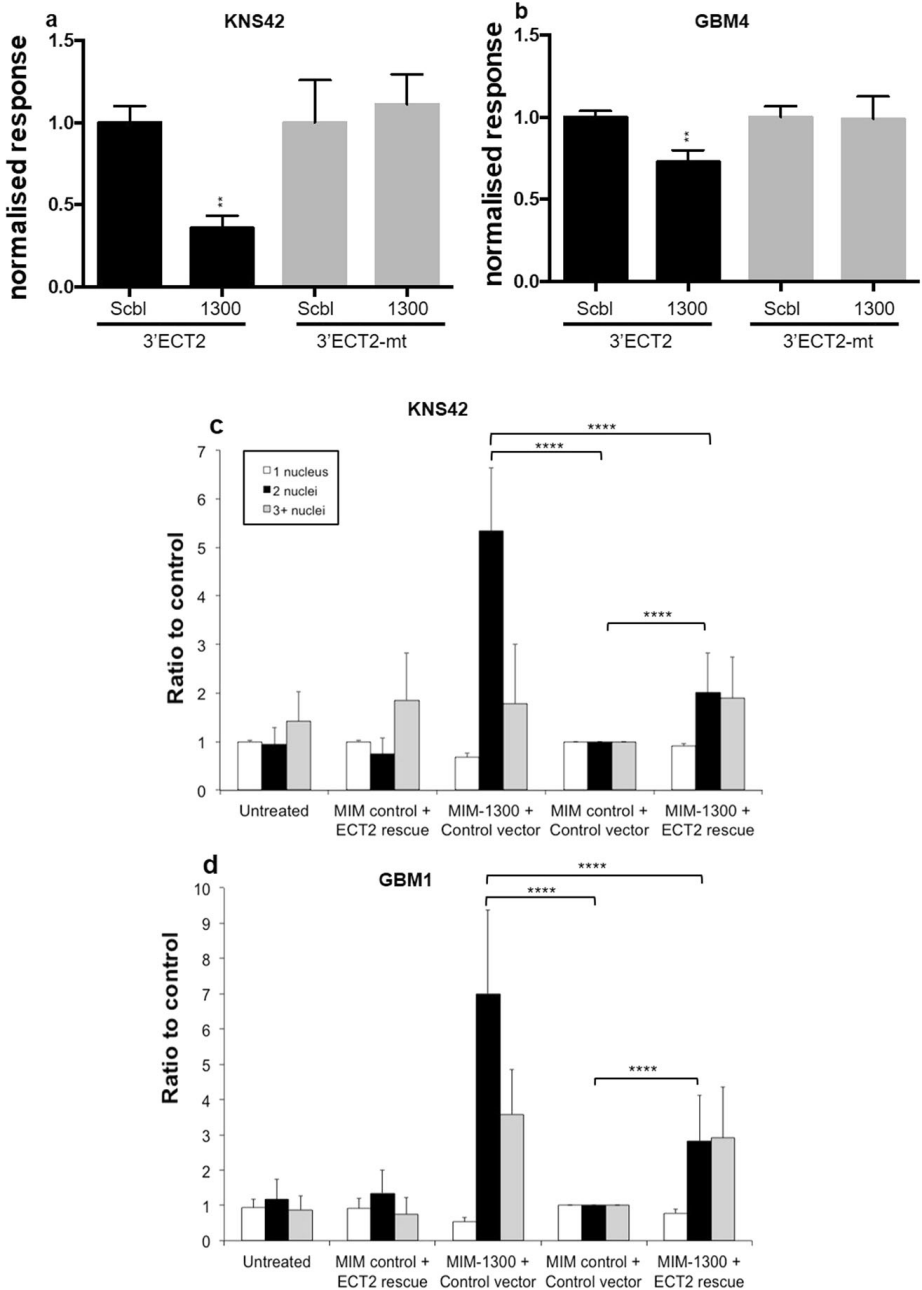
ECT2 is a direct target of miR-1300. Direct targeting assessed by 3′UTR luciferase assay in the KNS42 cells (**a**) and GBM4 GSCs (**b**). 3′ECT2 represents the wild-type 3′UTR sequence, 3′ECT2-mt represents the 3′UTR sequence containing two point mutations in the predicted binding site for the miR-1300 seed sequence. Ectopic expression of ECT2 rescues miR-1300 induced cytokinesis failure. Binucleation phenotype scoring (3–6 FOV per condition, representing at least 100 cells) was performed on images taken on the Operetta imaging platform (×10 objective) in KNS42 (**c**) and GBM1 (**d**). All experiments were performed in triplicate. The results are normalized to the double control: “MIM control + Control vector” representing the scrambled-mimic combined with the empty vector devoid of the ECT2 expression cassette. Statistical significance is expressed as follows: **p < 0.01 and ****p < 0.0001.

Having shown that the miR-1300 phenotype is consistent across a range of established and patient-derived GBM lines and that *ECT2* is a promising target of miR-1300, direct targeting was confirmed using 3′UTR reporter assays. Cells were transfected with a luciferase reporter containing either the wild-type *ECT2* 3′UTR (3′ECT2) region or a mutated version of the *ECT2* 3′UTR (3′ECT2-mt) harboring two point mutations in the predicted miR-1300 seed region. Co-transfection of either KNS42 or GBM4 with miR-1300 and 3′ECT2 caused a significant reduction in reporter signal (Fig. 5a, b, respectively). In cells transfected with 3′ECT2-mt the effect of miR-1300 on the reporter signal was abolished thus showing that the *ECT2* 3′UTR is a direct target of miR-1300 (Fig. 5a, b, respectively).

In order to further validate the involvement of *ECT2* in the pathway downstream of miR-1300, we performed rescue experiments as follows; 36 h following transfection with miR-1300 in KNS42 cells (~60 h in GBM1 cells), cells were transfected again with an expression vector for *ECT2* (lacking the 3′UTR region) in an attempt to rescue cells from cytokinesis failure. We showed that re-expression of ECT2 caused a 50% reduction in the number of binucleated cells in both KNS42 and GBM1 cells (Fig. 5c, d). Overall, these data confirm that the effect of miR-1300 expression is mediated via reduced ECT2 levels, which drive failed cytokinesis and apoptosis in glioma cells.

### MiR-1300 and ECT2 as regulators of endomitosis

**Fig. 6:**
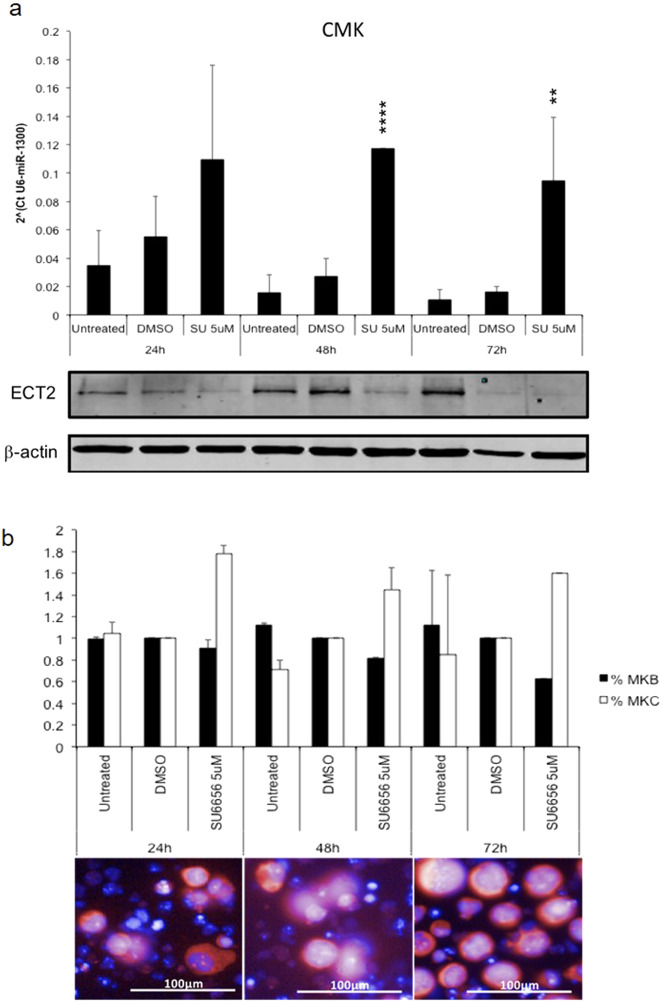
Exposure of synchronized CMK cells to SU6656 concomitantly induces an increase in the levels of miR-1300 and a decrease of its target, ECT2. **a** Cells were synchronized using Monastrol 25 mM for 24 h prior to exposure to SU6656 5 mM. **b** Increase in multinuclear megakaryocytic cells (MKC) as a result of endomitosis undergone by megakaryoblastic cells (MKB) was measured by immunofluorescence in response to SU6656. Cells were stained with DAPI and TOTO-3. Nine fields of view were analyzed using an algorithm designed in the Columbus software to discriminate objects based on their size (**b**). Statistical significance is expressed as follows: *p < 0.05; **p < 0.01; ***p < 0.001; ****p < 0.0001.

Endogenous levels of *ECT2* mRNA and protein are known to be decreased during megakaryocytic differentiation at the endomitotic stage when multinucleation occurs [19]. This suggests a likely regulatory role for miR-1300 on *ECT2* expression levels in platelet formation which is supported by low levels of miR-1300 remaining in platelets post terminal differentiation (see Supplementary data of Ref. [15]) [19, 42]. Using the megakaryocytic cell (MKC) line CMK [43] and the Src inhibitor SU6656 which induces endomitosis and differentiation of CMK cells into platelets [43, 44], these phenotypes were confirmed and this model of induced endomitosis utilized to validate this previously undescribed physiological role of miR-1300. We measured a time dependent increase in expression of endogenous microRNA-1300 in CMK cells by approximately fourfold at 48 h, and approximately sixfold at 72 h and a concomitant decrease in ECT2 protein levels following exposure to 5 μM SU6656 (Fig. 6a and Supplementary Fig. 8). Moreover, we confirmed the increase of polyploid MKC by nearly twofold at 24 h, and 1.5-fold both at 48 h and 72 h, respectively, using high-content immunofluorescence imaging (Fig. 6b).

### Differentiated brain tumor cells are not affected by miR-1300 expression

In order to establish whether the phenotype caused by miR-1300 was specific to proliferating GBM cells, we assessed the effect of its ectopic expression on fully differentiated GSCs, compared with their stem-like, proliferative (isogenic) counterparts. Differentiation of GBM1 and GBM4 cells was achieved by 5 days exposure to BMP4 as previously described [32, 33, 45–47]. First, we confirmed that BMP4 exposure significantly decreases proliferation in miR-1300 transfected cells (Supplementary Fig. 9).

**Fig. 7:**
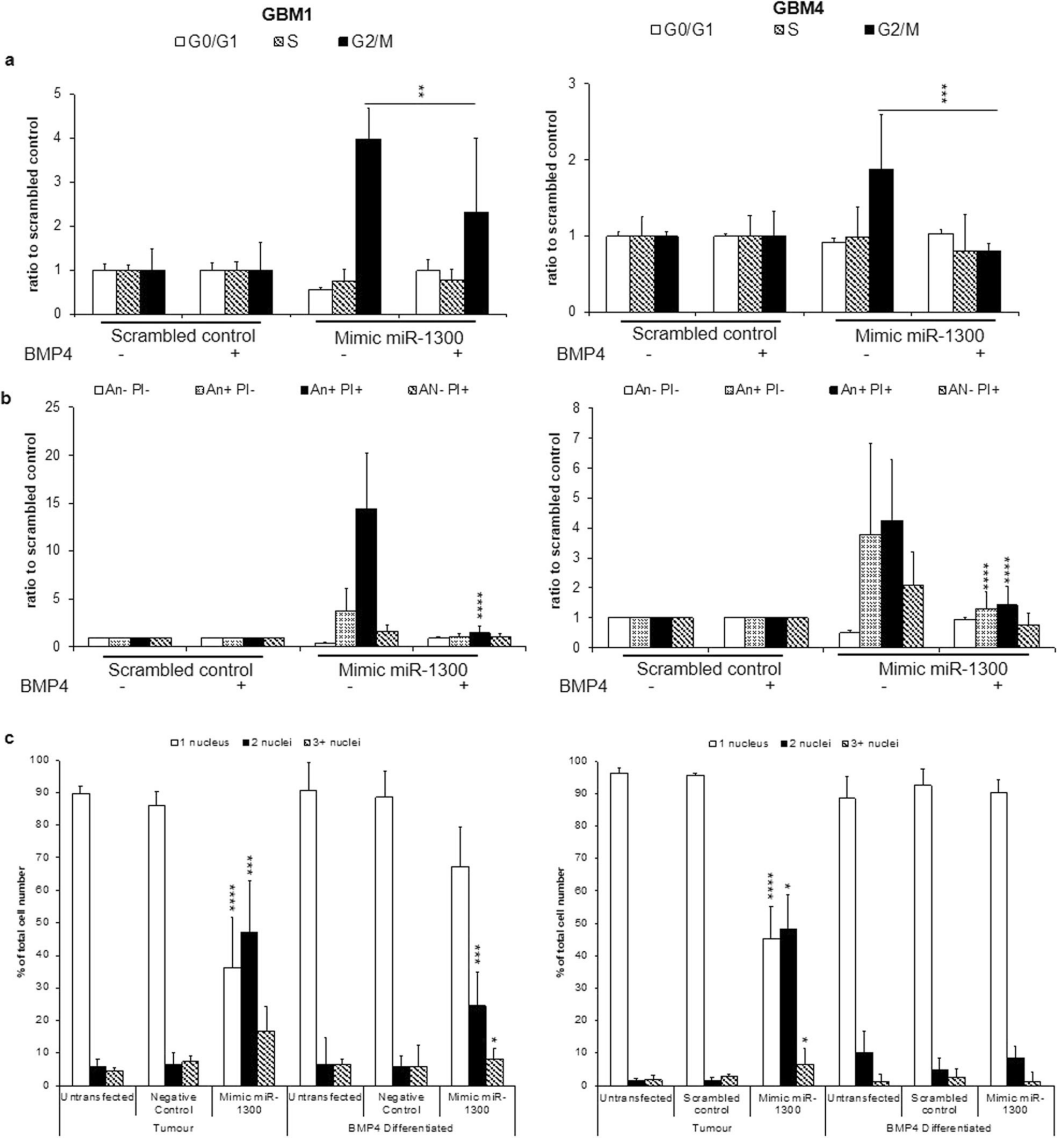
Ectopic expression of miR-1300 specifically affects GBM stem-like cells but not their differentiated counterparts. **a** Effect on the cell cycle was analyzed by flow cytometry 120 h post transfection using Propidium Iodide (PI) loading as a measure of DNA content. **b** Cell death measured also 120 h post transfection by flow cytometry where Annexin negative (An−), PI negative (PI−) = live cells, An+ PI− = early apoptotic cells, An+ PI+ = mid/late apoptotic cells and An− PI + = necrotic cells. **c** Binucleation phenotype scoring (3–6 images per condition, representing at least 100 cells) was performed on images taken on the Operetta imaging platform (×10). All experiments were performed in triplicate. The results are normalized to a scrambled-mimic control without BMP4 exposure. Statistical significance is expressed as follows: *p < 0.05; **p < 0.01; ***p < 0.001; ****p < 0.0001.

In GBM1 cells, transfection with miR-1300 caused a fourfold increase in the proportion of cells in G2/M phase, which was significantly reduced by 2.3-fold in the BMP4 differentiated counterparts (Fig. 7a). MiR-1300 caused a 1.5-fold reduction in live cell numbers, a 3.8-fold increase in early apoptotic cells and a 14-fold increase in apoptotic cells, whereas there was no significant change in their differentiated counterparts (Fig. 7b). A reduction by half in the number of binucleated cells in BMP4 differentiated GSCs compared with nondifferentiated cells was observed.

GBM4 cells showed a 0.8-fold increase in cells in G2/M after transfection with miR-1300, which was reduced to a 0.2-fold after BMP4 treatment (Fig. 7a). MiR-1300 caused a 50% reduction in live GBM4 cells and a 3.8- and fourfold increase in early-mid apoptotic and mid-late apoptotic cells, respectively. However, cell death was significantly reduced in BMP4 treated postmitotic GBM4 cells transfected with miR-1300, to the point where there were no significant differences in comparison with the scrambled control transfected cells (Fig. 7b). BMP4 treated GBM4 cells did not show an increased number of binucleated cells compared with control cells, as opposed to their nondifferentiated counterparts (Fig. 7c). Taken together these data suggest that expression of miR-1300 in nonproliferating, differentiated cells does not induce significant cell cycle arrest or apoptosis when compared with proliferating, undifferentiated (stem-like) isogenic pairs. This suggests that miR-1300 could represent an attractive target with a favorable therapeutic rational in GBM.

### Ectopic expression of miR-1300 decreases tumor growth in an orthotopic GBM mouse model

**Fig. 8:**
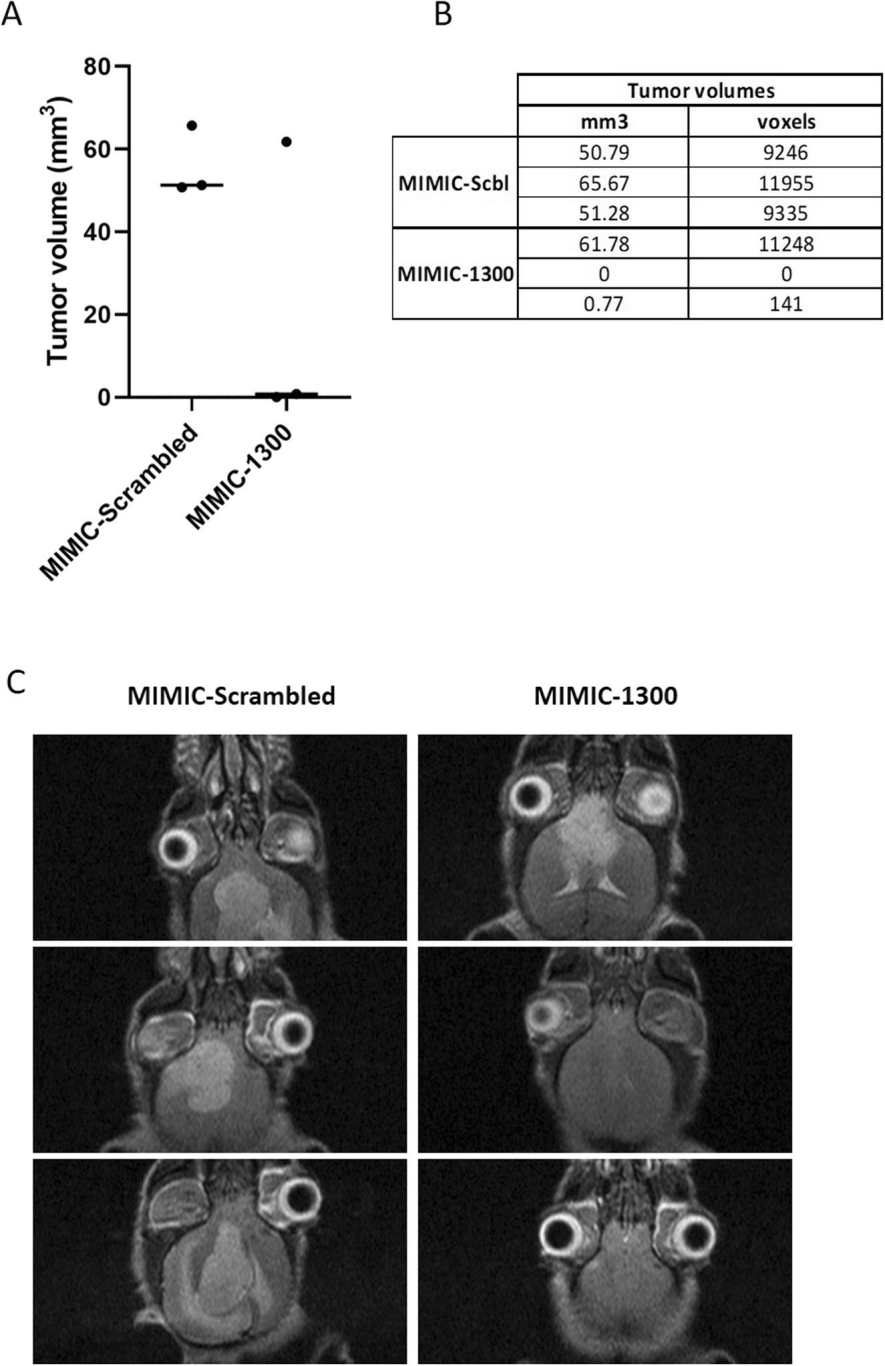
Ectopic expression of miR-1300 impairs tumor growth in an orthotopic GBM mouse model. **a** Tumor volumes plotted in GraphPadPrism 8. **b** Tumor volumes analyzed using VivoQuant software. **c** Representative MRI images for each animal in the study. Images were acquired using a 7T MRI System (AspectImaging, Watford, UK), using a T2 weighed fast spin echo scan for 10 min.

To assess the effect of miR-1300 in a preclinical mouse GBM model we reverse-transfected U87 cells with miR-1300 or a negative control microRNA (Scrambled), as before. Cells remained in culture for 2½ days to maintain balance between transfection efficiency and cell death due to the miR-1300 phenotype. Three animals per group received an intracranial implantation of transfected U87 cells. Tumor volume was measured by MRI at day 26 upon the first sign of neurological symptoms, showing a decrease in tumor volume in the miR-1300 group in comparison to controls (Fig. 8). Of note, the MRI images in Fig. 8c shows that the larger tumor in the miR-1300 group (top right image) displays some structural differences compared with the control tumors of equivalent volumes. The contrast in this tumor is less than that of the control tumors and it edges of the tumor are also more diffuse. Thus, miR-1300 transfection results in a demonstrable alteration in tumor growth in vivo and will be explored in further studies.

## Discussion

GBM is an aggressive brain tumor with no effective treatment [48]. MicroRNAs are well-known as important regulators of cellular functions [3–5] and several have been shown to have therapeutic potential, either by overexpression or inhibition by antisense oligonucleotides. Thousands of microRNAs are encoded in the human genome, therefore, to investigate which microRNAs may have effects on GBM growth and to identify novel sensitivities and potential therapeutic approaches we performed a high-throughput screen of a microRNA library comprising all known human microRNAs (miRBase v16). Our high-throughput high-content screen defined the landscape of microRNAs with significant cytotoxic effects in adult and pediatric GBM cell lines. We identified over 100 microRNAs with significantly potent effects in our screen, which are fully disclosed in this paper. The results of this study should therefore provide a major resource for the GBM field, as many of these microRNAs are yet unstudied in GBM or other contexts. We validated our top hits in an extended panel of GBM-derived cells and based on overall potency and induction of apoptotic cell death we followed up in detail the effects of miR-1300. Ectopic expression of miR-1300 after transfection of oligonucleotide mimics leads to a distinctive phenotype of cytokinesis failure followed by apoptosis in both established GBM cell lines and in patient-derived GBM cultures. Prior to their death, cells acquire a distinctive bi-/multinucleated phenotype due to cytokinesis failure. We used bioinformatics analysis followed by functional analyses to identify and validate ECT2 as a direct target and critical effector downstream of miR-1300. Ect2 is a Rho-GEF with known functions in cell division [40–44].

MicroRNAs are frequently altered in cancer and represent bona fide clinical agents particularly when applied in combination with effective viral or nanoparticle delivery systems. Clinical trials are underway in liver cancer and multiple microRNA-based strategies are in development for the treatment of cancer [8]. Our next series of experiments will employ viral vectors to deliver miR-1300 in vivo. Here, we focused on detailed studies of in vitro cell killing and cellular phenotypes, showing a remarkably robust defect in cytokinesis. Our pilot in vivo data shows that miR-1300 effects are reflected in tumor growth in an orthotopic GBM model, supporting further therapeutic studies.

MiR-1300 has not previously been studied in terms of its biological function. Our investigations led us to find that miR-1300 has an important physiological function in megakaryocyte differentiation, in which platelets are formed by punching-off from a multinucleated megakaryocyte parental cell in the bone marrow [19]. This fascinating observation relates to its function in GBM cell killing and reveals that microRNAs with nontumor related physiological functions may be applicable in the treatment of disease, particularly cancer.

The susceptibility of GBM to cell cycle blockade should be further explored. We are currently investigating whether miR-1300 alone or in combination with other microRNAs or siRNAs expressed as an artificial miR Cluster as described by Bhaskaran et al. [11] will be a more effective inducer of cell cycle blockade and cell death. Indeed, intratumoral delivery of these multiple microRNA expressing constructs can be much more effective than expressing a single microRNA and offer a further advantage of exosomal intercellular spread throughout the tumor microenvironment.

The effect of miR-1300 shows its translational potential as a treatment for GBM. It also may be a very promising candidate for combination therapy as a chemo-radiosensitizer decreasing the ability of dividing cells to recover from the damage induced by conventional therapy. Moreover, this effect could be of paramount use in the second line of treatment whereby specifically targeting resistant repopulating cells it could also significantly impair recurrence.

Bioinformatics revealed additional targets beyond ECT2 of miR-1300, which may impact and reinforce the phenotype we observed. Some of these are under further investigation. Thus, our ongoing work is aimed at validating the mechanism of action of miR-1300 in more molecular detail as well as addressing delivery of microRNAs for brain tumor treatment and its potential use in combination with conventional treatments.

## Materials and methods

Please see Supplementary information for: cell line details, and additional methods.

### High-throughput screen

The miRIDIAN microRNA MIMIC library based on miRBase v16.0 was purchased from Dharmacon (GE Healthcare). All microRNA mimics and siRNAs were resuspended at a stock concentration of 20 μM in 1× siRNA Buffer prepared from a 5× stock (Cat# B-002000-UB-100, Dharmacon GE Healthcare) in RNase free water (Cat# B-003000-WB-100, Dharmacon GE Healthcare). The screen controls for cell number were MIMIC negative control #1 tagged with Dy547 (Cat# CP-004500-01-20) to calculate transfection efficiency as well as without Dy547 (Cat# CN-001000-01-20), which were included in eight separate wells and PLK-1 siRNA SMARTpool (Cat# M-003290-01-005), which was both the transfection control and the positive control for decreased cell number. Reverse transfection was performed on U251 and KNS42 cells using RNAiMAX lipofectamine (Invitrogen, Life Technologies, Cat# 13778-075), OPTI-MEM^®^ I Reduced Serum Medium (Cat# 31985, Gibco™, Invitrogen corporation) and 100 nM of RNA material (MIMIC or siRNA candidates and controls). For the screen, each well of a 96-well ViewPlate (6005182, Perkin Elmer), contained 20 μl of transfection mix including: 0.1 μl of RNAiMAX (137789-075, Life Technologies) commercial stock solution, 0.1 μl of 20 μM microRNA, and 19.8 μl of OptiMEM^®^ media (31985-047, Life Technologies); to which 80 μl of growth media, containing 6 × 10^3^ cells/well (U251) or 9 × 10^3^ cells/well (KNS42), were added giving a final volume of 100 μl per well. Plates were placed at 37 °C, 5% CO_2_ for 72 h prior to being fixed in 4% PFA and stained with DAPI (Cat# D1306, Life Technologies) for nuclei count, TOTO-3 (Cat# T-3604, Molecular Probes) and Phalloidin-A488 (Cat# A12379, Life Technologies) for delimitation of the cytoplasm and actin structure.

Each 96-well plate contained one microRNA mimic or microRNA control per well. Plates were imaged on a Perkin-Elmer Operetta High-Content Imaging System using Harmony 3.1 software, three fluorescent channels, ten-fields/well with a 20× objective. For each cell line, the screen was performed in two separate passages of cells.

An algorithm for nuclei counting based on DAPI stain was designed in Columbus 2.4 analysis software. For the analysis, the mean cell number of eight mimic negative control wells was evaluated per plate and used to calculate the *Z*-score (the number of standard deviations above or below the mean cell number) for each mimic microRNA utilized. Candidate microRNAs were identified as those which significantly decreased cell number due to reduced proliferation and/or cell survival only if their mean *Z*-score was greater or equal to two standard deviations below the mean *Z*-score of the negative controls for both biological replicates.

### Induction of endomitosis

Endomitosis was induced by culturing nonadherent CMK cells for up to 72 h in the presence of 5 μM of the Src kinase inhibitor SU6656 (Sigma Aldrich) [43, 44]. Six milliliters of cell suspension were harvested every day for 3 consecutive days. Two milliliters were used to prepare RNA (SU6656 experiments only), 2 ml to prepare protein lysates for ECT2 western blot, and 2 ml were used for imaging.

### Forced differentiation of glioma stem cells

Glioma stem cells were differentiated in the presence of 100 ng/ml BMP4 [32, 33, 47] (Life Technologies) for 4–5 days prior to transfection with mimic miRNA-1300 or scrambled control. Cells were maintained in BMP4 supplemented media following transfection.

### Preclinical model

U87 cells were transfected either with a nontargeting mimic-scrambled or our mimic-miR-1300 at 100 nM as described in the screen conditions. Cells were then harvested 63 h post transfection in order to achieve the best balance between induction of the miR-1300 phenotype and cell death as per our in vitro data.

Animal handling procedures and experiments were performed in accordance with the UK Animal Scientific Procedures Act 1986 and covered by UK Home Office licenses (University of Leeds ethics committee project license:PA67C4EBE4). All animals are housed in Tecniplast green line caging in a pathogen free environment, given 3R’s bedding made from sterile recycled sterile paper material and fed a diet of compound rat mouse (Special Diets Services Ltd) and reverse osmosis filtered water.

At day 0, 1 × 10e5 viable U87 cells were orthotopically implanted by intracranial surgery into three Balb C Nude mice per cohort (Scrambled and miR-1300). Intracranial injection co-ordinates were 1 mm rostral to bregma, 1.5 mm lateral (right), and 4 mm deep.

Animals were then weighed and checked daily. MRI images were taken on the day of the first animal showing neurological symptoms which is this experiment fell on day 26 post surgery. MRI data were acquired using a 7T MRI System (AspectImaging, Watford, UK), using a T2 weighed fast spin echo scan for 10 min. Images were analyzed using VivoQuant software.

### Sample size and statistical analysis

With the exception of the high-throughput screen, all assays were performed as three biological replicates (cells from different passage number), each containing three technical replicates. In the case of immunofluorescent imaging analysis, a minimum of 100 cells per condition were scored. The unequal variance Welch’s, unpaired *t*-test was chosen to test how far apart the two populations tested were regardless of the difference in their standard deviation (for example: microRNA-1300 vs control).

## Supplementary information

Supplementary material

Supplementary Table 1

Supplementary Table 2

Supplementary Movie 1A

Supplementary Movie 1B

## Data Availability

The raw data from the screen are supplied in the supplementary information as individual Excel files.
